#  Mepolizumab: Which condition benefits more: Nasal polyps or eosinophilic asthma? 

**DOI:** 10.5578/tt.2025011058

**Published:** 2025-03-24

**Authors:** Elif AÇAR, Serpil KÖYLÜCE, Serhat ŞEKER, Hatice Eylül BOZKURT YILMAZ, Elif AKTAŞ YAPICI, Bahar ARSLAN, Gülden PAÇACI ÇETİN, Murat TÜRK, İnsu YILMAZ

**Affiliations:** 1 Division of Allergy and Clinical Immunology, Department of Chest Diseases, Erciyes University Faculty of Medicine, Kayseri, Türkiye; 2 Division of Allergy and Clinical Immunology, Kayseri City Hospital, Kayseri, Türkiye; 3 Division of Allergy and Clinical Immunology, Kocaeli City Hospital, Kocaeli, Türkiye

## Abstract

**ABSTRACT**

** Mepolizumab: Which condition benefits more: Nasal polyps or eosinophilic asthma?
**

**Introduction:** *The negative effects of eosinophilic severe asthma and chronic rhinosinusitis with nasal polyps (CRSwNP) on quality of life are well known. The present study aimed to evaluate the impact of mepolizumab treatment on the quality of life of patients diagnosed with eosinophilic severe asthma and CRSwNP *

**Materials and Methods:** *Patients with CRSwNP and eosinophilic severe asthma who had been receiving mepolizumab 100 mg/month for at least six months as treatment were eligible for the study. Patients were assessed using quality of life and disease control questionnaires for both diseases before and during the sixth month of treatment. *

**Results:** *Forty-nine patients were included in the present study. Compared to the pre-treatment period, there was significant clinical improvement in quality of life for both rhinitis and asthma following mepolizumab treatment. The changes before and after the treatment were found as follows: Rhinitis Quality of Life Scale [44 (23-72); 25 (6-57); p< 0.001], Nasal Congestion Symptom Assessment Scale [13.0 (8-20); 9.0 (0-20); p< 0.001], Sino-Nasal Outcomes Test-22 (63.16 ± 17.9; 44.16 ± 21.33; p< 0.001), Asthma Quality of Life Questionnaire (111.06 ± 25.17; 142.67 ± 28.04; p< 0.001), Asthma Control Test [16 (6-21); 22 (14-25); p< 0.001]. Before mepolizumab treatment, 20 patients defined that CRSwNP and 29 patients defined that asthma affected their quality of life primarily. After six months of mepolizumab treatment, 38 patients defined that CRSwNP and 11 patients defined that asthma affected their quality of life primarily *

**Conclusion:** *Our results showed that mepolizumab is an effective treatment option for eosinophilic severe asthma and CRSwNP in patients having both diseases. Before mepolizumab, most patients’ quality of life was affected by asthma symptoms, but this changed, and their quality of life began to be affected primarily by CRSwNP symptoms. This suggests that mepolizumab treatment is more effective in suppressing asthma symptoms than suppressing nasal polyp symptoms. *

**Key words:** *Type-2 inflammation; eosinophilia; chronic rhinosinusitis nasal polyposis; severe asthma; mepolizumab *

**ÖZ**

** Mepolizumab: Hangi durum daha fazla fayda sağlar: Burun polipleri mi, eozinofilik astım mı? **

**Giriş:** *Hem eozinofilik ağır astımın hem de nazal polipli kronik rinosinuzitin (KRS/NP) yaşam kalitesi üzerine olan olumsuz etkileri bilinmektedir. Bu çalışmada mepolizumab tedavisinin eozinofilik ağır astım ve KRS/NP tanılı hastalardaki yaşam kalitesi üzerine olan etkilerinin değerlendirilmesi amaçlanmıştır. *

**Materyal ve Metod:** *Çalışmada en az altı ay boyunca 100 mg/ay mepolizumab ile tedavi edilen KRS/NP ve eozinofilik ağır astım tanısı ile takip ettiğimiz hastalar incelendi. Hastalar, biyolojik tedavi öncesinde ve tedavinin altıncı ayında her iki hastalık için yaşam kalitesi ve hastalık kontrol anketleri kullanılarak değerlendirildi. *

**Bulgular:** *Çalışmamıza 49 hasta dahil edildi. Hastalarımızda mepolizumab tedavi sonrası rinit yaşam kalitesi ve astım yaşam kalitesinde tedavi öncesine göre belirgin klinik iyileşme saptandı. Tedavi öncesi ve sonrası değişimler; Rinit Yaşam Kalite Ölçeği [44 (23- 72); 25 (6-57); p< 0.001], Burun Tıkanıklığı Semptom Değerlendirme Ölçeği [13.0 (8-20); 9.0 (0-20); p< 0.001), Sino-Nasal Sonuç Test-22 (63.16 ± 17.9; 44.16 ± 21.33; p< 0.001), Astım Yaşam Kalite Anketi (111.06 ± 25.17; 142.67 ± 28.04; p< 0.001), Astım Kontrol Testi-22 [16 (6-21); 22 (14-25); p< 0.001]. Mepolizumab tedavisinden önce, 20 hasta KRS/NP’nin, 29 hasta astımın yaşam kalitesini daha olumsuz etkilediğini belirtirken, altı aylık mepolizumab tedavisi sonrası ise 38 hasta KRS/NP’nin, 11 hasta astımın yaşam kalitelerini olumsuz etkilediğini belirtti.
*

**Sonuç:** *Mepolizumabın her iki hastalığa sahip hastalarda eozinofilik şiddetli astım ve KRS/NP için etkili bir tedavi seçeneği olduğunu gösterdi. Mepolizumab öncesi hastaların çoğu yaşam kalitelerinin öncelikle astım semptomlarından dolayı etkilendiğini belirtmekteyken mepolizumab tedavisi sonrası bu durum değişmiş ve ön planda yaşam kaliteleri KRS/NP semptomları yüzünden etkilenmeye başlamıştır. Bu sonuç mepolizumab tedavisinin astım semptomlarını baskılamakta KRS/NP semptomlarını baskılamaktan daha etkin olduğunu düşündürmektedir *

**Anahtar kelimeler:** *Tip-2 inflamasyon; eozinofili; kronik rinosinüzit nazal polipozis; ağır astım; mepolizumab *

## INTRODUCTION

Chronic rhinosinusitis can be seen in 5-12% of the general population with two different phenotypes; one with nasal polyps (CRSwNP) and one without nasal polyps (CRSnNP). CRSwNP is seen in approxi- mately 1.1-4.3% of the population, and concomitant asthma may be present in 30-70% of them (1). Although CRSwNP is seen in 10-30% of mild asth- matics, this number might increase up to 40-90% in patients with severe asthma (2-4).

Accompanied by asthma, CRSwNP is called T2 NP, in which type-2 (T2) inflammation predominates. Eosinophilic inflammation is present in this patient phenotype (5,6). CRSwNP asthma, which forms a characteristic subgroup of late-onset eosinophilic asthma, has been known for many years. Some cases are associated with aspirin and other non-steroidal anti-inflammatory drug (NSAID) hypersensitivity (7).

The impacts of eosinophilic severe asthma and CRSwNP on quality of life were addressed in several studies before. Indeed, both diseases have been shown to have serious negative impacts on quality of life (8-10). In cases with both diseases, quality of life can be expected to be much more affected.

Mepolizumab is a humanized IgG1/k-class monoclo- nal antibody that binds to interleukin-5 (IL-5), pre- venting its interaction with IL-5 receptor alpha (IL-5R*α*) (11). Mepolizumab treatment has been shown to improve symptom scores and quality of life in eosinophilic severe asthma and CRSwNP (12-14). However, no investigation has been conducted about the extent to which mepolizumab treatment sepa- rately improves the quality of life in eosinophilic severe asthma with CRSwNP where both diseases coexist, and the extent to which quality of life ques- tionnaires in patients receiving the treatment revealed greater improvement for one disease over the other Thus, the present study aimed to compare the quality of life before mepolizumab and in the sixth month of treatment in this specific asthma phenotype.

### MATERIALS and METHODS

**Study Design and Patients**

Patients followed with diagnoses of CRSwNP and eosinophilic severe asthma that were treated with mepolizumab treatment between 2022 and 2023 were evaluated and those who met the study criteria were included in the study. Patients included in the study were older than 18 years of age, had difficult

asthma differentiated from severe asthma according to Global Asthma Initiative (GINA) criteria, had a confirmed diagnosis of eosinophilic severe asthma, had an accompanying diagnosis of CRSwNP con- firmed by endoscopic and/or nasal CT, and had not undergone surgery for NP in the last year and during the study period. In line with current guideline rec- ommendations, nasal saline irrigation, high-dose intranasal corticosteroids, and short-term systemic corticosteroids, if needed, were used for CRSwNP. For severe asthma, patients were treated with high- dose inhaled corticosteroids (ICS) and inhaled long- acting beta-agonists, as well as montelukast and/or long-acting anti-antimuscarinics, as needed, in line with the recommendations of the GINA. Mepoli- zumab was administered subcutaneously at doses of 100 mg every four weeks in patients with eosino- philic severe asthma whose asthma could not be controlled under step five treatment. Those with a severe active autoimmune disease, active malignan- cy, pregnancy, non-polyp chronic sinonasal disease, and another chronic lung disease accompanying asthma were excluded from the study.

Demographic and disease-related characteristics of the patients, nonsteroidal anti-inflammatory drug sensitivity and atopy status, family history, pulmonary function tests, laboratory data, and radiological imaging were obtained from medical records. Patients were evaluated with quality of life questionnaires before biological treatment and in the 6th month of treatment and were given Sino-Nasal Outcome Test- 22 (SNOT-22) and Nasal Congestion Symptom Evalu- ation Scale (NOSE) to evaluate rhinitis symptoms along with Rhinitis Quality of Life Scale (RQLS). The Asthma Control Test (ACT) was used to evaluate asthma symptoms and control status, and the Asthma Quality of Life Questionnaire (AQLQ) was used to evaluate asthma-related quality of life. In addition, the impacts of patients’ asthma and CRSwNP quality of life before and after mepolizumab treatment were evaluated comparatively with the Visual Analog Scale (VAS).

### Evaluation Scales

-
  The RQLS is the most commonly used specific scale to evaluate the quality of life of patients with rhinitis and consists of 26 questions and six sections (activity limitation, nasal symptoms, eye symptoms, sleep problems, symptoms outside

the eye and nose, general problems). Patients were asked to answer the questions by consider- ing their complaints in the last 15 days and rating their symptoms between 0 and 3 points (0: None,

1: Mild, 2: Moderate, 3: Severe). A low score indicates that rhinitis symptoms are good and a high score indicates that they are bad (15,16).

-
  The SNOT-22, the most commonly used tool for patient-reported chronic rhinosinusitis out- comes, was also used in the study. The tool rates 22 different symptoms associated with rhinologi- cal, ear, facial, general, physical, and psycho- logical areas, from 0 (no problem) to 5 (problem as bad as possible). Scores range from 0 to 110, with higher scores indicating severity of symp- toms (17). A change of eight or more points represents the minimum significant value (18).
-
  The Nasal Congestion Symptom Assessment Scale consists of five questions that evaluate nasal symptoms. Each question is asked to be given a score from 0 to 4 on the scale. A low score indicates good nasal functions and a high score indicates poor nasal functions (19).
-
  The ACT is a five-item questionnaire validated to evaluate asthma control. Each item is scored on a 5-point Likert form from 1 to 5 (1: Worst, 5: Best), with the total score defining uncontrolled (5-19) and well-controlled (20-25) asthma,

respectively (20).

-
  The AQLQ is a disease-specific 32-item ques- tionnaire designed to measure the functional impairments that are most distressing for adults with asthma. Patients are asked to remember their experiences over the past two weeks and score each item on a seven-point scale, with a higher score indicating fewer symptoms. The overall AQLQ score is the average response to all 32 questions (21).
-
  In the Visual Analogue Score, symptoms are scored from 1 to 10, with 0 points meaning no complaints and 10 points meaning the worst complaints (22).

Informed consent form was obtained from our patients along with the questionnaire form for study participation.

### Statistical Analysis

Statistical analysis was performed using SPSS version

22.0 (IBM Corp., Armonk, NY, USA). Categorical variables were expressed as frequencies and percent- ages, and the chi-square test was used to compare the groups. Normality of the data was analyzed by the Kolmogorov-Smirnov test. Non-normally distrib- uted data were expressed as median (minimum- maximum) and compared with the Mann-Whitney U test, and normal distributed data were expressed as mean ± standard deviation (SD) and compared with the independent sample t-test.

## RESULTS

A total of 49 patients (35 females and 14 males) who were started on 100 mg mepolizumab treatment

every four weeks were included in the study. Mean age of the patients was 47.3 ± 12.9 years, 28 (57.1%) had NSAID hypersensitivity, and 51% were oral corticosteroid-dependent (Table 1).

When the patients were evaluated again six months after mepolizumab treatment, we found that there was a significant decrease in the blood eosinophil count compared to the baseline values (935.9 ± 520.9 vs. 89.51 ± 69.7, p< 0.001). In the sixth month, the number of OCS-dependent patients declined significantly (51% vs. 18.4%, p< 0.001). Aside from this, a significant decrease was detected in the number of asthma exacerbations in the last six months when compared to the baseline value (83.7% vs. 12.2%, p< 0.001) (Table 2).

**Table:** 

**Table 1.** Demographic characteristics of the patients before biological treatment	
**Demographic Characteristics**	**Patients, n= 49**
Mean age ± SD	47.3 ± 12.9
Female sex, n (%)	35 (71.4)
Asthma phenotype, n (%)	
Eosinophilic+non-atopic	36 (73.4)
Eosinophilic+atopic	13 (26.6)
Aeroallergen sensitivity, n (%)	13 (26.5)
NSAID sensitivity, n (%)	28 (57.1)
OCS dependent, n (%)	25 (51)
Patients with an exacerbation in the previous year, n (%)	41 (83.7)
Blood eosinophil count, (mean ± SD), cells µL	935.9 ± 520.9
FEV1, mean ± SD, mL	2477.1 ± 812.27
FEV1, mean ± SD, (%)	85.4 ± 19.7
FEV1/FVC, mean ± SD	70.6 ± 7.8
Number of nasal polyp surgeries, median (min-max)	1.35 (0-7)
Presence of sense of smell, n (%)	14 (28.6)
FEV1: Forced expiratory volume in one second, FEV1/FVC: Forced expiratory volume in one second/Forced vital capacity, NSAID: Non-steroidal anti-inflammatory drug, OCS: Oral corticosteroid, SD: Standard deviation, Min-Max: Minimum-maximum.	

**Table:** 

**Table 2.** Patients’ data before and after biological treatment			
	**pre-Treatment Period**	**In the Sixth Month of Treatment**	**p**
OCS addiction, n (%)	25 (51)	9 (18.4)	<0.001
Eosinophil count, (mean ± SD), cells µl	935.9 ± 520.9	89.51 ± 69.7	<0.001
Patients with an exacerbation in the previous year, n (%)	41 (83.7)	6 (12.2)	<0.001
FEV1, mean ± SD, mL	2477.1 ± 812.27	2546.9 ± 846.4	0.010
FEV1, mean ± SD, (percent)	85.4 ± 19,7	87.8 ± 19.6	0.207
FEV1/FVC, mean ± SD	70.6 ± 7.8	71.3 ± 7.8	0.016
FEV1: Forced expiratory volume in one second, FEV1/FVC: Forced expiratory volume in one second/Forced vital capacity, OCS: Oral corticosteroid, SD: Standard deviation.			

**Table:** 

**Table 3.** Questionnaires for CRSwNP and severe asthma			
	**Baseline**	**In the Sixth Month of Treatment**	**p**
SNOT-22	63.16 ± 17.9	44.16 ± 21.33	<0.001
NOSE	13.0 (8-20)	9.0 (0-20)	<0.001
RQLS	44 (23-72)	25 (6-57)	<0.001
VAS-CRSwNP	8 (3-10)	4 (0-10)	<0.001
ACT	16 (6-21)	22 (14-25)	<0.001
AQLQ	111.06 ± 25.17	142.67 ± 28.04	<0.001
VAS-ASTHMA	8 (3-10 )	3 (0-7)	<0.001
ACT: Asthma control test, AQLQ: Asthma quality of life questionnaire, NOSE: Nasal congestion symptom evaluation, RQLS: Rhinitis quality of life scale, SNOT-22: Sino-nasal outcome test-22, VAS: Visual analogue scale.

Considering the effects of mepolizumab treatment on rhinitis symptoms, when compared to baseline scores, SNOT-22 (63.16 ± 17.9 vs. 44.16 ± 21.33,

p< 0.001), NOSE [13.0 (8-20) vs. 9.0 (0-20), p<

0.001] and RQLS [44 (23-72) vs. 25 (6-57), p< 0.001]

scores were significantly decreased after six months of treatment. Baseline CRSwNP visual analog scores were a median of 8 (3-10) points and the scores decreased after treatment to 4 (0-10) points (Table 3).

When considering the effects of mepolizumab treat- ment on asthma symptoms, the baseline ACT increased from 16 (6-21) to 22 (14-25) points (p< 0.001). Similarly, after six months of mepoli- zumab treatment, a significant improvement was detected in the AQLQ (111.06 ± 25.17 vs. 142.67 ± 28.04, p< 0.001). We also observed a more than 50% decline in asthma visual analog scores (Table 3).

Another main aim of our study was to determine which disease affects patients’ quality of life and to compare the trend after mepolizumab treatment. When patients were asked which of these two dis- eases primarily affected and had a more negative impact on their quality of life, 20 (41%) patients reported that CRSwNP and 29 (49%) patients report- ed that severe asthma had a more negative impact on their quality of life at baseline, and 38 (78%) patients reported that CRSwNP and 11 (22%) patients report- ed that severe asthma had a more negative impact on their quality of life in the sixth month.

## DISCUSSION

Severe asthma and CRSwNP severely impact patients’ quality of life, both separately and together. Although CRSwNP is not a disease that can cause mortality like severe asthma, it reduces the quality of life seriously (23,24). In the present study, after six

months of mepolizumab treatment, significant clini- cal improvement was detected in all CRSwNP and asthma scores in patients having both of these dis- eases. We also showed that while asthma was the most disruptive disease at baseline, it was shifted to CRSwNP in the sixth month of mepolizumab treat- ment.

It was confirmed by many real-life studies that mepolizumab reduces the number of exacerbations in eosinophilic severe asthma, decreases OCS dependence, and improves the quality of life (25,26). Furthermore, the positive impacts of mepolizumab on post-treatment SNOT-22, VAS symptom scores, radiological imaging, and quality of life (RQLQ) in T2 NP were demonstrated in real-life studies. Significant improvements were detected in the quality of life measured by RQLS. Similarly, in the questionnaires conducted for NP, in line with the literature data, a significant improvement was detected in the RQLS, SNOT-22, and NOSE questionnaires after the treat- ment compared to baseline, and a significant improvement was detected in the quality of life evaluated with VAS (27,28).

The most striking result of the study was that although most patients reported that their quality of life was more affected by asthma before mepolizumab, in the sixth month of treatment, most of the patients report- ed that their quality of life was more affected by CRSwNP. This shows that mepolizumab has positive effects on the quality of life of both disease groups, which is similar to some other studies (25-29). Moreover, its positive impacts on the quality of life in asthma are more dominant, for this reason, the nega- tive impacts of NP on the quality of life come to the fore in relative terms after treatment.

Moreover, in the present study, when the patients were asked which disease affected the quality of life more in eosinophilic NP patients before mepoli- zumab treatment, the rate of those who answered severe asthma was high (59.18%), but in the sixth month of mepolizumab treatment, the rate of those who answered NP to the same question was found to be higher (77.55%). This shows that the positive impacts of mepolizumab on quality of life are more significant in severe eosinophilic asthma than in NP. Although not identical in methodology to our study, Chan et al. also reported that, in patients with CRSwNP, mepolizumab treatment initiated for eosin- ophilic severe asthma led to a positive response in asthma control, while the same positive effect was not observed for nasal polyps (30).

Although a significant improvement in clinical and quality of life was detected with mepolizumab in both diseases, the detection of a more significant improvement in quality of life in asthma suggests that the effect of mepolizumab treatment on inflamma- tion in asthma is more effective, although this is speculative. This shows the negative impact of the other comorbid diseases on the quality of life when the quality of life in one disease improves more sig- nificantly. For this reason, more effective treatments on the quality of life in eosinophilic severe asthma with NP, at least as much as in asthma, may lead to similar improvements in the quality of life of both diseases and provide a significant improvement in the overall quality of life. Despite being hypothetical, this can be achieved with biological combinations such as anti-IgE, anti-IL5/IL-5R, and anti-IL-4R. However, studies with these combination treatments are needed in this CRSwNP eosinophilic severe asthma phenotype to achieve this.

One of the most important limitations of the present study was that there was no specific validated quality of life questionnaire used for CRSwNP. On the other hand, since pre- and post-treatment evaluations were made with RQLS, SNOT-22, and VAS, these test results might provide information about the change in quality of life. However, in this respect, there is a need for a validated standard patient-reported outcome measure that evaluates NP quality of life. Another limitation was that mepolizumab treatment was start- ed in all patients in the study because of the indica- tion of severe asthma, and the systemic steroid needs during follow-up were determined and adjusted

according to asthma control. After mepolizumab, systemic steroids could be discontinued or the dose reduced in most patients. This might have caused an increase in patients’ nasal polyp symptoms. Another limitation of our study is that although the impact of mepolizumab on the quality of life for both nasal polyps and eosinophilic severe asthma was objec- tively assessed using quality of life surveys, the com- parison of its effects on the quality of life of both dis- eases was evaluated subjectively. However, since we could not compare the quality of life survey results for the two separate diseases, we had to make a subjec- tive comparison based on the patients’ assessments.

In conclusion, mepolizumab treatment improves clinical and quality of life outcomes in patients with CRSwNP and eosinophilic severe asthma, and the improvement in quality of life in asthma was more significant. For this reason, the negative effect of CRSwNP on quality of life has become relatively more apparent. In this context, there is a need for treatment modalities to improve all aspects of the quality of life in eosinophilic asthma accompanied by CRSwNP.

### Acknowledgment

We would like to thank the Proofreading & Editing Office of the Dean for Research at Erciyes University for the copyediting and proofreading service for this manuscript. We would like to thank Chiesi pharma- ceutical company for their editorial support.

**Ethical Committee Approval:** This study was approved by Erciyes University Faculty of Medicine Clinical Research Committee (Decision no: 2023/498, Date: 09.08.2023).

### CONFLICT of INTEREST

The authors declare that they have no conflict of interest.

## AUTHORSHIP CONTRIBUTIONS

Concept/Design: İY, EA, SK, SŞ, HEBY, EAY, GPÇ, MT

Analys/Interpretation: İY, EA, SK, SŞ, HEBY, EAY, GPÇ, MT

Data acqusition: İY, EA, SK, SŞ, HEBY, BA, GPÇ, MT Writing: İY, EA, SK, SŞ, EAY, BA, MT

Clinical Revision: İY, EA, SK, SŞ, BA, GPÇ, MT Final Approval: All of authors

## REFERENCES

1. *Fokkens WJ, Lund V, Bachert C, Mullol J, Bjermer L, Bousquet J, et al. EUFOREA consensus on biologics for CRSwNP with or without asthma. Allergy 2019; 74(12): 2312-9. https://doi.org/10.1111/all.13875*
2. *Canonica GW, Malvezzi L, Blasi F, Paggiaro P, Mantero M, Senna G, et al. Chronic rhinosinusitis with nasal polyps impact in severe asthma patients: Evidences from the Severe Asthma Network Italy (SANI) registry. Respir Med 2020; 166: 105947. https://doi.org/10.1016/j.rmed.2020.105947*
3. *Langdon C, Mullol J. Nasal polyps in patients with asthma: Prevalence, impact, and management challenges. J Asthma Allergy 2016; 9: 45-53. https://doi.org/10.2147/JAA.S86251*
4. *Philpott CM, Erskine S, Hopkins C, Kumar N, Anari S, Kara N, et al. Prevalence of asthma, aspirin sensitivity and aller- gy in chronic rhinosinusitis: Data from the UK National Chronic Rhinosinusitis Epidemiology Study. Respir Res 2018; 19(1): 129. https://doi.org/10.1186/s12931-018-0823-y*
5. *Akdis CA, Bachert C, Cingi C, Dykewicz MS, Hellings PW, Naclerio RM, et al. Endotypes and phenotypes of chronic rhinosinusitis: A PRACTALL document of the European Academy of Allergy and Clinical Immunology and the American Academy of Allergy, Asthma & Immunology. J Allergy Clin Immunol 2013; 131(6): 1479-90. https://doi.org/10.1016/j.jaci.2013.02.036*
6. *Fokkens WJ, Lund VJ, Hopkins C, Hellings PW, Kern R, Reitsma S, et al. European position paper on rhinosinusitis and nasal polyps 2020. Rhinology 2020; 58(Suppl S29): 1-464. https://doi.org/10.4193/Rhin20.601*
7. *Licari A, Brambilla I, De Filippo M, Poddighe D, Castagnoli R, Marseglia GL. The role of upper airway pathology as a co-morbidity in severe asthma. Rev Respir Med 2017; 11(11): 855-65. https://doi.org/10.1080/17476348.2017.1381564*
8. *Mullol J, Azar A, Buchheit KM, Hopkins C, Bernstein JA. Chronic rhinosinusitis with nasal polyps: Quality of life in the biologics era. J Allergy Clin Immunol Pract 2022; 10(6): 1434-53.e9. https://doi.org/10.1016/j.jaip.2022.03.002*
9. *Bachert C, Hellings PW, Mullol J, Hamilos DL, Gevaert P, Naclerio RM, et al. Dupilumab improves health‐related quality of life in patients with chronic rhinosinusitis with nasal polyposis. Allergy 2020; 75(1): 148-57. https://doi.org/10.1111/all.13984*
10. *Penezić A, Paić M, Gregurić T, Grgić MV, Baudoin T, Kalogjera L. The impact of asthma on quality of life and symptoms in patients with chronic rhinosinusitis. Curr Med Res Opin 2020; 36(6): 1043-8. https://doi.org/10.1080/03007995.2020.1754189*
11. *Gallo S, Castelnuovo P, Spirito L, Feduzi M, Seccia V, Visca D, et al. Mepolizumab improves outcomes of chronic rhinosinusitis with nasal polyps in severe asthmatic patients: A multicentric real-life study. J Pers Med 2022; 12(8): 1304. https://doi.org/10.3390/jpm12081304*
12. *Demir M, Tunakan Dalgic C, Mete Gokmen EN, Savas R, Eroglu S, Ozden G, et al. The effectiveness of biological agents on chronic rhinosinusitis with nasal polyposis in patients with comorbid asthma: A multicenter real-life study from Türkiye. Medicina 2024; 60(3): 448. https://doi.org/10.3390/medicina60030448*
13. *Han JK, Bachert C, Fokkens W, Desrosiers M, Wagenmann M, Lee SE, et al. Mepolizumab for chronic rhinosinusitis with nasal polyps (SYNAPSE): A randomised, dou- ble-blind, placebo-controlled, phase 3 trial. Lancet Respir Med 2021; 9(10): 1141-53.https://doi.orhttps://doi.org/10.1016/S2213-2600(21)00097-7*
14. *Bravo-Gutiérrez FJ, Miralles-López JC, Valverde-Molina J, Alemany Francés ML, Andújar-Espinosa R, Castilla- Martínez M, et al. Effectiveness of mepolizumab in patients with severe eosinophilic asthma with/without nasal polyposis: A real-life study. Int Arch Allergy Immunol 2024; 185(3): 253-9. https://doi.org/10.1159/000534902*
15. *Adadan GI, Ecevit MC. Kronik rinit ve rinosinüzitte subjek- tif değerlendirme yöntemleri: Yaşam kalitesi ölçekleri. Turkish J Rhinology 2016; 5(1); 38-52. https://doi.org/10.24091/trhin.2016-52685*
16. *Özşeker F, Büyüköztürk S, Gelincik A, İşsever H, Erdenen F, Özşeker H, et al. Bir Türkçe rinit yaşam kalitesi ölçeği geliştirme ve geçerlilik çalışması. Nobel Med 2012; 8(2): 32-40.*
17. *Cakir Cetin A, Kumus O, Keskinoglu P, Sutay S, Ecevit MC. Turkish validation of the sino‐nasal outcome test‐22. Clin Otolaryngol 2019; 44(4): 557-64. https://doi.org/10.1111/coa.13332*
18. *Hopkins C, Gillett S, Slack R, Lund V, Browne J. Psychometric validity of the 22‐item sinonasal outcome test. Clin Otolaryngol 2009; 34(5): 447-54. https://doi.org/10.1111/j.1749-4486.2009.01995.x*
19. *Spataro E, Most SP. Measuring Nasal Obstruction Outcomes. Otolaryngol Clin North Am 2018; 51(5): 883-95. https://doi.org/10.1016/j.otc.2018.05.013*
20. *Nathan RA, Sorkness CA, Kosinski M, Schatz M, Li JT, Marcus P, et al. Development of the asthma control test: A survey for assessing asthma control. J Allergy Clin Immunol 2004; 113(1): 59-65. https://doi.org/10.1016/j.jaci.2003.09.008*
21. *Juniper EF, Guyatt GH, Epstein RS, Ferrie PJ, Jaeschke R, Hiller TK. Evaluation of impairment of health related qual- ity of life in asthma: Development of a questionnaire for use in clinical trials. Thorax 1992; 47(2): 76-83. https://doi.org/10.1136/thx.47.2.76*
22. *Gevaert P, Omachi TA, Corren J, Mullol J, Han J, Lee SE, et al. Efficacy and safety of omalizumab in nasal polyposis: 2 randomized phase 3 trials. J Allergy Clin Immunol 2020; 146(3): 595-605. https://doi.org/10.1016/j.jaci.2020.05.032*
23. *Alobid I, Benítez P, Bernal-Sprekelsen M, Guilemany JM, Picado C, Mullol J. The impact of asthma and aspirin sen- sitivity on quality of life of patients with nasal polyposis. Qual Life Res 2005; 14: 789-93. https://doi.org/10.1007/s11136-004-1597-x*
24. *Lange B, Holst R, Thilsing T, Baelum J, Kjeldsen A. Quality of life and associated factors in persons with chronic rhi- nosinusitis in the general population: A prospective ques- tionnaire and clinical cross‐sectional study. Clin Otolaryngol 2013; 38(6): 474-80. https://doi.org/10.1111/coa.12189*
25. *Bulut İ, Katran ZY, Yavuz D, Akyıldız A, Yakut T, Örçen C, et al. Effectiveness of mepolizumab in patients with severe eosinophilic asthma: In a real life study. Tuberk Toraks 2023; 71(2): 148-55. https://doi.org/10.5578/tt.20239918*
26. *Li H, Zhang Q, Wang J, Gao S, Li C, Wang J, et al. Real- world effectiveness of mepolizumab in severe eosinophilic asthma: A systematic review and meta-analysis. Clinical Therapeutics 2021; 43(6): e192-e208. https://doi.org/10.1016/j.clinthera.2021.03.023*
27. *Huang CC, Wang CH, Fu CH, Huang CC, Chang PH, Chen IW, et al. The link between chronic rhinosinusitis and asthma: A questionnaire-based study. Medicine (Baltimore) 2016; 95(31): e4294. https://doi.org/10.1097/MD.0000000000004294*
28. *Domínguez-Sosa MS, Cabrera-Ramírez MS, Marrero- Ramos MdC, Dávila-Quintana D, Cabrera-López C, Carrillo-Díaz T, et al. Real-life effectiveness of mepolizum- ab in refractory chronic rhinosinusitis with nasal polyps. Biomedicines 2023; 11(2): 485. https://doi.org/10.3390/biomedicines11020485*
29. *Bakakos P, Alobid I, Constantinidis J, Hellings P, Pfaar O, Taillé C, et al. A response to anti-IL-5 therapy in comorbid patients with chronic rhinosinusitis with nasal polyps and severe asthma: Study protocol. J Allergy Clin Immunol Glob 2024; 4(1): 100343. https://doi.org/10.1016/j.jacig.2024.100343*
30. *Chan Rory, Chris RuiWen Kuo, Brian Lipworth. Disconnect between effects of mepolizumab on severe eosinophilic asthma and chronic rhinosinusitis with nasal polyps. J Allergy Clin Immunol Pract 2020; 8(5): 1714-6. https://doi.org/10.1016/j.jaip.2020.01.009*

